# Why do married women procure abortion? Experiences from Ile-Ife, south western Nigeria

**DOI:** 10.4314/ahs.v21i1.42

**Published:** 2021-03

**Authors:** Ibitola Eunice Ojo, Temitope Olumuyiwa Ojo, Ernest Okechukwu Orji

**Affiliations:** 1 Primary Health Center, Enuwa, Ile-Ife, Family Planning Unit; 2 Obafemi Awolowo University College of Health Sciences, Department of Community Health; 3 Obafemi Awolowo University College of Health Sciences, Department of Obstetrics, Gynaecology and Perinatology

**Keywords:** Induced abortion, unwanted pregancies, married women, Nigeria

## Abstract

**Background:**

In Nigeria, about 1.25million induced abortions occur annually and the country accounts for one-fifth of abortion-related deaths globally.

**Objectives:**

The study aimed to assess the determinants of induced abortion among married women.

**Methods:**

A mixed methods study was conducted in Ile-Ife, Nigeria. The quantitative component employed a cross-sectional study design while the qualitative aspect comprised focus group discussions. Information on contraceptives use, unintended pregnancy and induced abortion were obtained from 402 married women (with at least one child) aged 18–49 years using a semi-structured questionnaire. Four focus group discussion sessions were conducted among women of reproductive age.

**Results:**

Majority (67.2%) of respondents had ever used a contraceptive method. However, 34.3% of the women have had unintended pregnancies and 14.2% had induced abortion. FGD findings revealed that non-use of contraceptives and contraceptive failure were major reasons for unintended pregnancies and induced abortion. The significant predictors of induced abortion were non-use of contraceptives, age≥ 40 years and multiparity.

**Conclusion:**

Induced abortion still occur among married women particularly those not using contraceptives, aged ≥40 years and those with high parity. More emphasis should be placed on making contraceptives more accessible to married women.

## Introduction

Globally, it has been estimated that 25 million unsafe abortions occur annually with 97% taking place in developing countries.^1^ Unsafe abortion is a leading cause of maternal mortality 2, 3 and most abortion-related deaths occur in low income countries.^4^ In Nigeria, about 1.25 million induced abortions take place each year with majority of them being unsafe. 5 Maternal mortality ratio in Nigeria is 545 per 100,000 live births^6^ and unsafe abortion is responsible for 30–40% of the maternal mortality.^7^ In Sub-Saharan Africa and indeed Nigeria, induced abortion is illegal but women often procure abortion clandestinely from unhygienic places or quacks.^5^,^8^ Acute complications of unsafe abortion may include haemorrhage, pelvic sepsis, septicaemia, renal shutdown, cervical laceration, uterine perforation, other genital tract injuries, gastro-intestinal tract injuries and death.^8^ Late complications comprise chronic pelvic inflammatory disease, ectopic pregnancy, secondary infertility, secondary amenorrhea, spontaneous abortion, and premature births.^9^

Increasing urbanization and the involvement of women in paid employment as well as the weakening capacity of families to cope with child bearing and rearing of many children have led to a desire for somewhat smaller families. 10 In addition, the natural methods of contraception that Nigerian women used to space births (postpartum abstinence and exclusive breast-feeding) are on the decline, particularly among working women. Also, a considerable proportion of Nigerian women in the reproductive age are not using modern contraceptives.^6^ The aforementioned factors may contribute to a higher burden of unwanted pregnancy that may end up in induced abortion. Despite a slow increase in the proportion of married women using modern methods of contraception in Nigeria - from 7% in 2003 to 12% in 2018,^6^ recent observations from clinical practice showed an increasing trend of hospitalization of married women in Ile-Ife due to complications of induced abortion. Oye-Adeniran et al reported that married women constituted 30.2% of abortion seekers in a study carried out in south western Nigeria.^11^ Bankole et al documented that married women resort to abortion to meet their child bearing goals such as spacing or limiting births.^12^ It has also been documented that it is not only adolescents and single women that procure induced abortion, married women also engage in the practice for socio- economic and personal reasons.^13^ The inability of family planning programmes to meet the contraceptive needs of married women who are at risk of unintended pregnancies may underline the high rates of unwanted pregnancies and induced abortion in Nigeria. Previous research work has shown that married women contributed significantly to the overall burden of induced abortion but there is need for more knowledge on the factors responsible for unwanted pregnancy that may result in induced abortion amongst them. The current prevalence of induced abortion needs to be determined as the last published study on the prevalence of induced abortion in Ile-Ife was in 1996.^14^ This is important because the frequency of induced abortion is an important indicator of the availability and use of contraceptives.^15^ This study assessed the prevalence and factors associated with induced abortion among married women in Ife Central Local Government. The findings from the study will be useful in repositioning family planning programmes to meet the needs of married women.

## Methods

A mixed methods survey was conducted in Ife Central Local Government, Ile-Ife, Osun State, Nigeria. The quantitative method was a cross-sectional survey while the qualitative method involved focus group discussion. The study population comprised 402 currently married women aged 18 to 49 years who have at least one living child in Ife Central Local Government. The respondents were selected for this study through a two-staged sampling technique. Five health facilities out of 12 were selected using simple random sampling technique. The selected health facilities were: Urban Comprehensive Health Centre, (UCHC) Eleyele; Comprehensive Health Centre, Enuwa; Comprehensive Health Centre, Aderemi; Primary Health Centre, Ile Canaan; Primary Health Centre, Gbalefefe. The total number of surveyed respondents per facility was determined proportionately based on the average number of married women of reproductive age that visit the health facility per month. At each facility, respondents were recruited consecutively at the general out-patient department until the allotted number of respondents for the facility was complete. Currently married women who had at least one living child were included in the study. Women who had severe acute illnesses were excluded from the study. Data was collected over a period of 10 weeks between September and November, 2014.

**Quantitative method:** Data were collected using a semi-structured, interviewer-administered questionnaire on socio-demographic characteristics, reproductive health history and knowledge, information on unintended pregnancy and induced abortion. The questionnaire was translated into Yoruba and administered in English and Yoruba language as Ile-Ife is predominantly a Yoruba-speaking town. Interviewers comprised 5 students enrolled for the Master of Public Health program of the Obafemi Awolowo University and they were trained for 3 days before being deployed to the field.

**Qualitative method:** Four focus group discussion sessions were conducted among women of reproductive age group at UCHC, Eleyele and PHC, Ile-Canaan. Each group consisted of eight discussants who did not participate in the quantitative survey. This method was used to get in-depth information about the practice and perspectives of induced abortion among married women. Each session was conducted by a facilitator and records were taken by a note taker.

**Quantitative data:** Data collected were analysed using Statistical Product and Service Solutions (SPSS) version 20 software. Descriptive statistics was used to summarize the data. The frequency distribution of socio-demographic characteristics of the respondents was done. Univariate analysis was also used to determine the awareness of respondents about contraceptives; the use of contraceptives; desire to space and limit birth; reason for contraceptive choice; the proportion of women with unintended pregnancies and induced abortion.

Chi-square test was used to assess relationship between respondents' socio-demographic characteristic, history of previous pregnancy, use of contraceptives and the dependent variables (ever had induced abortion and ever had unwanted pregnancy). Chi-square statistics was also used to assess the relationship between fertility intention of respondents and their contraceptive use. A p-value of 0.05 was considered statistically significant. Multivariate binary logistic regression analysis was used to assess determinants of induced abortion. Recorded interviews from the FGD sessions were transcribed, coded and analyzed according to themes.

### Ethical considerations

Ethical approval for the study was obtained from the Ethics and Research Committee of Obafemi Awolowo University Teaching Hospital, Ile-Ife. Permission was also obtained from the Executive Secretary of Ife Central Local Government through the director of Primary Health Care Services. Detailed information about the study were explained to the respondents, participation was voluntary and written consent was obtained. All information gathered was kept confidential as participants were identified using only serial numbers on a password secured computer.

## Results

Socio-Demographic Characteristics of the Respondents are presented in Table 1

**Table 1:** Socio-demographic characteristics of respondents (N=402)

Characteristics	Frequency (N)	Percentage (%)
**Age in years**		
18–24	84	20.9
25–29	143	35.6
30–34	119	29.6
35–39	41	10.2
>40	15	3.7
**Religion**		
Christianity	313	77.9
Islam	88	21.9
Traditional	1	0.2
**Level of education**		
None	7	1.7
Primary	24	6.0
Secondary	245	60.9
Tertiary	115	28.6
Post-tertiary	11	2.7
**Occupation**		
Artisan	108	26.9
Trader	178	44.3
Farmer	4	1.0
Civil servant	23	5.7
Privately employed	45	11.2
Housewife	44	10.9
**Income**		
Less than 5000	129	32.1
5001–10000	132	32.8
10001–20000	74	18.4
20001–50000	53	13.2
Above 50000	14	3.5
**Duration of marriage (years)**		
≤ 1	66	16.4
2–4	138	34.3
5–9	107	26.6
≥10	91	22.6
**Number of Living Children**		
1	144	35.8
2	126	31.3
3	77	19.2
4	36	9.0
≥5	19	4.7

All participants (402) were currently married women who had at least one child attending the primary health facilities in Ife Central Local Government. Most respondents (86.1%) were less than 35 years and 60.9% of all respondents had secondary education. Majority (71.2%) of the respondents were artisans and traders while 10.9% were unemployed. About two thirds (64.9%) of respondents earned less than 10000 naira ($33) monthly (Central bank of Nigeria's official exchange rate was 306 Naira to 1 US dollar as at January 2020). About three quarters (77.4%) of the respondents had been married for less than 10 years. About one-third (35.8%) of respondents had one living child while 4.7% had 5 or more living children.

About two-thirds (67.2%) of the respondents had used contraceptives at any time prior to the survey. About one-third (34.3%) of the respondents reported that they had had unintended pregnancies. Among those with previous unintended pregnancies, 84.1% reported that they had only one unintended pregnancy. Overall, 14.2% of respondents reported that they had had induced abortion. Majority (84.8%) of respondents that had unintended pregnancies reported that they were not using contraceptives at the time they got pregnant while 15.2% got pregnant due to contraceptive failure. For those that reported contraceptive failure, the pills, natural method and emergency contraception had the higher rates of failure. About four out of ten respondents who had had unintended pregnancy attempted abortion and succeeded.

Dilatation and curettage was the method of induced abortion in 57.4% of cases. Majority (82%) of the induced abortions were done in private hospitals. Most cases (82%) of induced abortions were carried out by doctors and nurses while 16.4% of the respondents who had had abortion developed complications after the procedure. Among those with complications, 80% reported that they had excessive bleeding and 70% of them went to private hospitals for the treatment of the complication. The respondents with 5 or more living children were more likely to have had induced abortion than the respondents with lesser number of living children. The relationship between number of living children and induced abortion was statistically significant (p= 0.008). There was no statistically significant relationship between age, religion, level of education, occupation, income, marriage duration and induced abortion. The respondents who had never used contraceptives reported a higher rate of induced abortion but this relationship was not statistically significant.

Women aged 40 years or more were about 3 times more likely to have had induced abortion when compared with younger women aged 18–24 years. [OR 3.32, (95% C.I 1.09–11.54) p=0.048]. The respondents who had never used contraceptives were more likely to have had induced abortion as compared with those who had ever used contraceptives. [OR 2.38, (95% C.I 1.27–7.32) p=0.006].

Women who had 4 living children were 3 times more likely [OR 3.12, (95% C.I 1.97–17.89) p=0.008] to have had an induced abortion, those with 5 or more living children were about 7 times more likely [OR 6.91, (95% C.I 1.85–19.19) p=0.003] to have had an induced abortion as compared with those with one living child.

### Results from the qualitative study

Majority of the focus group discussion participants said that unintended pregnancies were fairly common among married women and that the major causes of unintended pregnancies were non-use of contraceptives, contraceptive failure and extra-marital affairs. Majority of the participants were aware that induced abortion is illegal in the country and that it is not an openly acceptable practice. Almost all participants believed that induced abortion could have health complications including death.

### Reasons for not using contraceptives, unintended pregnancy and induced abortion

Women had varied opinions about non-use of contraceptives ranging from ignorance to poor access, fear of side effects and non-approval from their husbands. A woman said: …“*some husbands are against the use of contraceptives as their belief is that a woman who uses contraceptive is having extra-marital affairs*”

Results from FGD revealed that a major reason for unintended pregnancy is the non-use of contraceptive use. For younger women it was mostly due to non-use of contraceptives while older women experienced more of contraceptive failure or pregnancy during the peri-menopausal period. A woman stated as follows: “*I had an experience, my previous child was already 10 years and I had decided not to have kids again. I had stopped having my menstrual period for 3 years. I thought I was having high blood pressure and went to the teaching hospital here in Ife, where they informed me that I was pregnant. When I got home, my husband and I decided to keep the pregnancy*”.

Women also believed that husbands are the major decision makers when it comes to procuring abortion. “*The husband might say that he is not ready for pregnancies at the particular time the woman got pregnant and they are usually the ones that makes the decision to terminate the pregnancies*.” Some reasons for procuring induced abortion as suggested by women includes economic reasons, to limit family size and extramarital affairs. “*Some women have extra-marital pregnancies and they terminate these pregnancies to prevent their husbands from getting to know”, A participant indicated that: “the reason husbands tell their wives to abort is mostly due to the financial circumstances. If there is no money to take care of an additional child, the next option is abortion*.”

## Discussion

### Prevalence of unintended pregnancy

Findings from the study showed that the rate of unintended pregnancies was very high among married women as 34.3% of respondents had had unintended pregnancies and 41.3% of these ended in abortion. This prevalence of unintended pregnancy was low when compared to the study carried out among currently married women in Ethiopia where the prevalence was 42.4%.^16^ The variation in prevalence might be because the Ethiopian study was carried out in a rural community while the Ile-Ife is a semi-urban community A rising trend in prevalence may be apparent considering the fact that the prevalence of unintended pregnancy among women of reproductive age was 19% in Ile Ife as at 1996. 14

Majority (82.6 %) of the women reported that they were not using contraceptive at the time they got pregnant. The fear of side effects was the major reason for nonuse of contraceptive as deduced from the FGDs. The side effects mentioned by respondents during the qualitative study included excessive weight gain, headaches, infections and excessive menstrual flow. Another reason given by some respondents was their husbands' disapproval. Contraceptives' failure was reported by 15.2% of respondents as the reason for previous unintended pregnancy and this is similar to findings from a survey in 8 Nigerian states where 16% of women were on modern contraceptives when they had an unwanted pregnancy.^17^ The respondents who were using pills reported the highest number of method failure and this might be due to incorrect or inconsistent use of the contraceptives.

This study also recorded that unintended pregnancy was more common amongst married women aged 18 to 24 years and those who were above 40 years. This might be due to the fact that majority of the young women got married because they were pregnant. Another reason for this might be the age at which the women got married as some studies have revealed that women who got married around 20 years of age had a higher likelihood of having unintended pregnancies when compared to the women who got married between 25 years and above.^16^, ^18^ The older women got pregnant when they did not want as most of them might have been experiencing menopausal symptoms and were not using any form of contraceptive. 16

### Prevalence of induced abortion

This study recorded a prevalence of 14.2% for induced abortion among married women. This showed a slight increase when compared with a prevalence of 10.5% reported by Okonofua and colleagues.^19^ However, while the denominator in our study was married women, the study by Okonofua et al included both single and married women. Findings from the focus group discussions showed that the reasons why women procure induced abortion ranges from financial constraints, short birth intervals to extramarital pregnancies, these reasons were similar to findings of other previous studies within and outside the country.^12^, ^16^, ^20^, ^21^ Majority of the abortions were done in private hospitals by health workers (doctors and nurses) and this was in consonance with previous studies that reported that a substantial proportion of induced abortions were carried out by health workers in private hospitals.^14^, ^22^

Post abortion complications occurred in 16.4% of cases and the common ones were pain and excessive bleeding. This report is similar to previous studies.^19^, ^22^ In addition, FGD findings revealed that infertility could be a common post-abortion complication. Majority of those who experienced post-abortion complication were treated at the private hospitals where they had the abortion though about 30% of them reported that they had self-medicated. This was in line with the study by Bankole et al which found that only one-third of women who developed post-abortion complications sought treatment.^12^ This was corroborated by the finding from the qualitative study where a woman noted thus: – “*Some of them will just stay at home and die rather than go to the hospital because they don't want anyone to know what they have done*”. This may suggest that women who procure induced abortion may feel stigmatised and delay seeking care if complications occur. The major reasons for abortion stigmatization may be due to social norms, religion and the country's legal statutes which are against abortion. It has been documented that morbidity and mortality arising from induced abortion is high in countries with restrictive abortion laws.^23^–^25^

### Factors associated with induced abortion

The predictors of induced abortion as determined by the binary logistic regression model were age, previous contraceptives use and number of living children. Women older than 40 years were more likely to have had induced abortion. This appears to be a national trend as evidence from a pooled analysis of the Nigeria demographic health survey from 2003 to 2013 corroborates this.^26^ A possible reason is that menopausal symptoms usually start around this period and women may not use contraceptives since they feel they are no longer at risk of pregnancy. Therefore, pregnancies that occur at this period may be unwanted and are more likely to be terminated through induced abortion. In contrast, a study conducted in Ibadan, Nigeria reported that younger women (20–29 years) were more likely to have had induced abortion. 27 This difference might be explained by the fact that the Ibadan study was conducted among antenatal clinic attendees and not married women as was the case in our study.

Married women who had never used contraceptives were more likely to have reported an induced abortion when compared with those who had ever used contraceptives. A similar finding was reported in the Nigeria demographic health surveys from 2003–2013 where evidence suggested that the use of contraceptives significantly reduces the likelihood of having an induced abortion. 26 A logical explanation for this is that sexual exposure without the use of contraceptives when pregnancy is not desired could lead to unwanted pregnancies and eventually induced abortion. Moreover it has been reported in previous studies that some women perceive induced abortion as a convenient way of child spacing and limiting.^21^, ^28^

In addition, findings from this study also showed that women with 4 or more children, had higher odds of having an induced abortion in comparison with their counterparts with one living child. This finding is similar to that of previous studies from Nigeria, Kenya and Bangladesh where women of higher parity were more likely to have had induced abortion.^26^, ^29^, ^30^ A plausible reason for this is that women with higher parity are more likely to have completed their desired family size. Any new pregnancy beyond their desired family size may be seen as unwanted, therefore actions may be taken to terminate such pregnancy.

Further research should entail a detailed country-wide study to assess social and cultural factors and their influence on induced abortion among married women. This will improve the understanding of the determinants of induced abortion among married women. In addition, studies employing wholly qualitative approaches will be essential in unraveling the social and cultural factors underlining induced abortion among married women.

A possible study limitation was that the prevalence of induced abortion might have been under-reported as the respondents may be unwilling to indicate that they had had induced abortion (social desirability bias), since it is illegal in Nigeria. However, this limitation was reduced to the barest minimum as the respondents were assured of confidentiality and interviews were conducted outside the home environment. In addition, this study was conducted in a semi urban town in Nigeria, therefore findings may not be generalizable to the entire country.

## Conclusion

This study showed that married women have a reasonably high rate of unintended pregnancy, induced abortion and unmet need for family planning despite a high level of awareness and availability of family planning methods. The study also found that due to the illegality of induced abortion in the country, women who resort to abortion to terminate unintended pregnancies patronise patent medicine vendors and other places where the procedure is unsafe. The findings from this study have implications for improving maternal health. Unintended pregnancy and induced abortion still occur among married women particularly those who do not use contraception, women aged 40 years or more and those with high parity. It is essential that married women are counselled appropriately on the importance of contraceptives use and its role in limiting and spacing childbirths. This would improve contraceptives uptake among them, thereby stemming the tide of unintended pregnancies and induced abortions.

## Figures and Tables

**Table 2:** Prevalence of unintended pregnancy and induced abortion

Characteristics	Frequency (N)	Percentage (%)
**Contraceptive ever used**		
Yes	270	67.2
No	132	32.8
Total	402	100
**Unintended pregnancies**		
Yes	138	34.3
No	264	65.7
Total	402	100
**Number of unintended pregnancies**		
1	116	84.1
2	14	10.1
3	6	4.3
4	2	1.4
Total	138	100.0
**Induced abortion**		
Yes	57	14.2
No	345	85.8
Total	402	100.0
**Number of Induced abortion**		
1	53	93.0
2	4	7.0
Total	57	100.0

**Table 3:** Reason for unintended pregnancy and actions taken by women

Reason for unintended pregnancy (n=138)	Frequency (N)	Percentage (%)
No contraceptive use	114	84.8
Contraceptive failure	21	15.2
Total	138	100
**Method used by women who had contraceptive failure (n=21)**		
Implants	1	4.8
Pills	6	28.6
IUCD	2	9.5
Natural method	5	23.8
Injectables	2	9.5
Emergency contraception	5	23.8
Total	21	100
**Action taken concerning the unintended pregnancy** **(n=138)**		
Did nothing	73	52.9
Attempted a removal but was unsuccessful	8	5.8
Attempted a removal and was successful	57	41.3
Total	138	100

**Table 4:** Method of induced abortion and pattern of post abortal complication

Method of induced abortion	Frequency (n=61)	Percentage (%)
Drugs	12	19.7
Vacuum aspiration	9	14.8
Dilatation and curettage	35	57.4
Injections	3	4.9
Traditional method	2	3.3
**Person who performed the abortion**		
Doctor	33	54.1
Nurse	17	27.9
Chemist	3	4.9
Self	2	3.3
Community health extension worker	5	8.2
Traditional attendants	1	1.6
**Place where abortion took place**		
Private hospital	50	82.0
Chemist	10	16.4
Traditional attendants	1	1.6
**Post abortion complication**		
Yes	10	16.4
No	51	83.6
Total	61	100.0
**Complication type**		
Abdominal pain	2	20.0
Excessive bleeding	8	80.0
Total	10	100.0
**Treatment of post abortion complication**		
Private clinic	7	70.0
Self-treatment	3	30.0
Total	10	100.0

**Table 5:** Association between socio-demographic characteristics and induced abortion

Characteristics	Ever had induced abortion= NO (n=345)		Ever had induced abortion=YES (n=57)		Statistical comparison
	N	%	N	%	
**Age of respondents**					
18–24 years	73	86.9	11	13.1	
25–29 years	128	89.5	15	10.5	χ^2^= 7.512
30–34 years	98	82.4	21	17.6	Df=4
35–39 years	36	87.8	5	12.2	P=0.111
≥40 years	10	66.7	5	33.3	
**Religion**					
Christianity	266	85.0	47	15.0	LR= 1.092
Islam	78	88.6	10	11.4	Df=2
Traditional	1	100	0	0	P=0.579
**Level of education**					
None	6	85.7	1	14.3	
Primary	23	95.8	1	4.2	χ^2^= 2.813
Secondary	206	84.1	39	15.9	Df=3
Tertiary	110	87.3	16	12.7	P=0.421
**Occupation**					
Housewife	43	97.7	1	2.3	
Trader	151	84.8	27	15.2	χ^2^= 8.958
Farmer	3	75.0	1	25.0	Df=4
Artisan	87	80.6	21	19.4	P=0.062
Civil servant	61	89.7	7	10.3	
**Income**					
Less than 5000 naira	110	85.3	19	14.7	
5001 – 10000 naira	114	86.4	18	13.6	χ^2^= 0.699
10001– 20000 naira	63	85.1	11	14.9	Df=4
20001– 50000 naira	45	84.9	8	15.1	P=0.938
Above 50000 naira	13	92.9	1	7.1	
**Marriage duration**					
1 year	54	81.8	12	18.2	
2–4 years	123	89.1	15	10.9	χ^2^= 3.083
5–9 years	93	86.9	14	13.1	Df=3
10 years and above	75	82.4	16	17.6	P=0.379
**Number of living children**					
1 child	120	83.3	24	16.7	
2 children	113	89.7	13	10.3	χ^2^= 13.697
3 children	71	92.2	6	7.8	Df=4
4 children	29	80.6	7	19.4	P=0.008
5 or more children	12	63.2	7	36.8	
**Ever used contraceptive**					χ^2^= 0.483
Yes	234	86.7	36	13.3	Df=1
No	111	84.1	21	15.9	P=0.487

**Table 6:** Binary logistic regression analysis of the association between respondents' characteristics and induced abortion

Variables	Odds ratio	95.0% C.I	P value
**Age in years**			
18–24	1.00	Ref	
25–29	0.78	0.34–1.78	0.551
30–34	1.42	0.65–3.13	0.382
35–39	0.92	0.30–2.85	0.888
≥40	**3.32**	**1.09–11.54**	0.048*
**Ever used contraceptive**			
Yes	1.00	Ref	
No	**2.38**	**1.27–7.32**	0.006*
**Number of living children**			
1 child	1.00	Ref	
2 children	1.25	0.40–2.68	0.659
3 children	2.00	0.765–6.194	0.738
4 children	**3.12**	**1.97–17.892**	0.008*
≥5 children	**6.91**	**1.85–19.19**	0.003*
